# First report of *Pratylenchus penetrans* (Nematoda: Pratylenchidae) associated with artichokes in Vietnam

**DOI:** 10.2478/jofnem-2023-0060

**Published:** 2024-01-23

**Authors:** Thi Duyen Nguyen, Huu Tien Nguyen, Thi Mai Linh LE, Quang Phap Trinh

**Affiliations:** Institute of Ecology and Biological Resources, Vietnam Academy of Sciences and Technology, 18 Hoang Quoc Viet, Cau Giay, 100000 Hanoi, Vietnam.; Graduate University of Science and Technology, Vietnam Academy of Sciences and Technology, 18 Hoang Quoc Viet, Cau Giay, 100000 Hanoi, Vietnam.

**Keywords:** D2-D3, Lam Dong, molecular, root-lesion nematode, systematics, taxonomy, 18S, 28S

## Abstract

*Pratylenchus penetrans* is one of the world’s most common and destructive root-lesion nematodes and can parasitize more than 400 plant species. *P. penetrans* has been reported to cause serious damage to artichokes in several countries, such as Greece, Brazil, and France. Until now, there have been no reports of *P. penetrans* associated with artichokes in Vietnam. In this study, we recorded this species in artichoke fields in Lam Dong province, Vietnam with an average density of 50 nematodes/100g of soil (frequency of appearance at 64.7%). This nematode was associated with symptoms such as yellowing leaves, stunt, and root necrosis of artichokes in Vietnam, indicating its high damaging potential and a need for suitable control strategies. The identification of this species in our study was confirmed by morphology, morphometric data, and molecular characterization of 18S and 28S rRNA regions. Our study also provides the first molecular data of *P. penetrans* in Vietnam. The inclusion of molecular data for *P. penetrans* in Vietnam represents a significant contribution to the scientific community and a pivotal advancement in addressing nematode-related challenges in agriculture. This dataset serves as an invaluable reference for various molecular-focused endeavors, including but not limited to molecular identification, pathogenicity studies, and the development of effective management strategies.

Root-lesion nematodes, particularly those in the genus *Pratylenchus*, are among the most economically significant plant-parasitic nematodes and can cause substantial damage to a variety of host plants (Castillo & Vovlas, 2007; Geraert, 2013; Sikora *et al.*, 2018). Currently, more than 103 species of the genus *Pratylenchus* have been described, each with its own host range and specific characteristics (Geraert, 2013; Nguyen *et al.*, 2019). *Pratylenchus penetrans* (Cobb, 1917; Filipjev & Schuurmans Stekhoven, 1941) is one of the most destructive species and has been reported on every continent except Antarctica (Castillo & Vovlas, 2007). Nearly 400 host plants can be infected by this species (Nyczepir & Becker, 1998). Its adverse impact on various crops, e.g. carrots, potatoes, tomatoes, and tobacco, has been extensively studied (Castillo & Vovlas, 2007; Sikora *et al.*, 2018). For example, Miller (1975) reported that a population density of 8–55 *P. penetrans*/100g of soil at planting reduced tomato plant growth by 20–66% after two months. In carrots, even an initial population density of 0.1 *P. penetrans*/cm^3^ of soil caused 75% of the carrots to be branched, and 1 nematode/cm^3^ killed 40% of the carrots (Coosemans, 1975). In Canada, infections of tobacco with *P. penetrans* resulted in significant economic losses, with population densities as low as six nematodes/1g of soil causing yield losses of 11%, and 18 nematodes/1g causing losses of 27.5% (Olthof *et al.*, 1973). *Pratylenchus penetrans* is a primary causal agent of replant problems in many parts of the world (Nyczepir & Becker, 1998). While the detrimental effects of *P. penetrans* on artichokes have been documented in several countries, such as Greece, Brazil, and France (Castillo & Vovlas, 2007; Caubel *et al.*, 1975; Rossi & Monteiro, 2001; Vovlas & Roca, 1981), its presence and impact in Vietnam, a significant producer of artichokes (particularly in Lam Dong, the most productive province in the country, where 162 hectares of artichokes are cultivated, yielding an impressive output of 8200 tons), remains unexplored to the best of our knowledge.

In Vietnam, *P. penetrans* was first reported in Lam Dong in 1988 based only on morphological characterizations (Nguyen & Nguyen, 2000). Currently, eight plant species, including potato (*Solanum tuberosum*), onion (*Allium fistulosum*), sugar beet (*Beta vulgaris*), cabbage (*Brassica oleracea* var. *capitata*), carrot (*Daucus carota*), sweet potato (*Impomoea batatas*), pea (*Phaseolus* sp.), and coffee (*Coffea arabica*), were recorded as hosts of *P. penetrans* (Nguyen & Nguyen, 2000). However, to the best of our knowledge, the identification of *P. penetrans* in Vietnam has never been confirmed by molecular data and has not been associated with artichokes until now. The identification of *Pratylenchus* species using morphological characters alone can be problematic due to their high interspecific similarity and intraspecific variability (Bogale *et al.*, 2021; Janssen *et al.*, 2017a; Janssen *et al.*, 2017b). Therefore, it is recommended to employ both molecular analysis and morphological observation to ensure more accurate identification of *Pratylenchus* species (Janssen *et al.*, 2017a).

## Materials and Methods

### Sampling and nematode extraction

After removing the debris layer, soil and root samples were obtained from the upper 25cm soil layer within the artichoke rhizosphere in Lam Dong, Vietnam using a shovel. The sampling was conducted randomly in the fields during the dry season (11/2022–07/2023). Specifically, a total of 34 samples were collected from 7 fields, with each field yielding 5 samples, except for the last field, which provided 4 samples. These samples were individually stored in nylon bags prior to transport to our laboratory for further extraction. To extract nematodes from collected samples, the modified Baerman tray method was used (Whitehead & Hemming, 1965). One nematode population was selected for detailed morphological and molecular taxonomical analyses. Nematodes from other samples were assessed morphologically using temporary slides to expedite the process while ensuring a representative analysis.

### Morphological characterization

The extracted nematodes were killed in hot water (60–70 ºC) for 30s before transferring to TAF solution for fixation for 4–5 days (Courtney *et al.,* 1955). Subsequently, nematodes were transferred to glycerin to make permanent slides following the method of Seinhorst (1959). Finally, nematodes were measured and photographed using a Carl Zeiss Axio Lab A1 microscope.

### Molecular characterization

For molecular characterization, each living nematode was cut into small pieces to enhance the efficiency of DNA extraction after collecting morphological vouchers. The resulting pieces were carefully transferred to a PCR tube containing 20 μl of worm lysis buffer (50 mM KCl; 10 mM Tris pH 8.3; 2.5 mM MgCl2; 0.45% NP-40 (Tergitol Sigma); 0.45% Tween-20). Ten nematodes were prepared for DNA extraction, and the sample was incubated at −20 degrees Celsius for at least 10 minutes, and 1 μl of proteinase K (1.2 mg ml^−1^) was added. The sample was then incubated in a PCR machine for 1 hour at 65°C and 10 minutes at 95°C before being centrifuged for 1 minute at 1400 rpm.

The D2-D3 of 28S rRNA and 18S rRNA regions were amplified using D2A/D3B (5′-ACAAGTACCGTGGGGAAAGTTG-3′/5′–TCGGAAGGAACCAGCTACTA–3′) and MN 18F/Nem_18S_R (5′-CGCGAATRGCTCATTACAACAGC-3′/5′-GGGCGGTATCTGATC GCC-3′) primers with the following thermal profile: 1 cycle of 94ºC for 4 min; 5 cycles of 94ºC for 30s; 56ºC for 30s; 72ºC for 2 min; 45 cycles of 94ºC for 30s; 54ºC for 30s; 72ºC for 1 min; and 10ºC for 10 min (Nguyen *et al.*, 2021a; Nunn, 1992). The Wizard SV Gel and PCR Clean-Up System from Promega, Madison, Wisconsin, USA, was used to purify all successful PCR reactions before sending them to Macrogen (Korea) for sequencing. In the next step, Geneious R11 (www.geneious.com) was used to assemble the obtained forward and reverse sequences. To find closely related sequences from GenBank, Blast search was employed (Altschul *et al.*, 1997). All selected sequences were aligned using MUSCLE, and the Bayesian phylogenetic analysis was performed using the MrBayes 3.2.6 add-in in Geneious R11. The best-fit models for the Bayesian phylogenetic analysis were selected using Mega 7 (Nguyen *et al.*, 2021b).

## Results

*Pratylenchus penetrans* was found in 64.7% of the 34 investigated soil samples taken from the growing area of artichokes in Vietnam with a mean density of 50 individuals/100g of soil in the positive samples.

### Morphological characterization

#### Female

**Figure 1.**
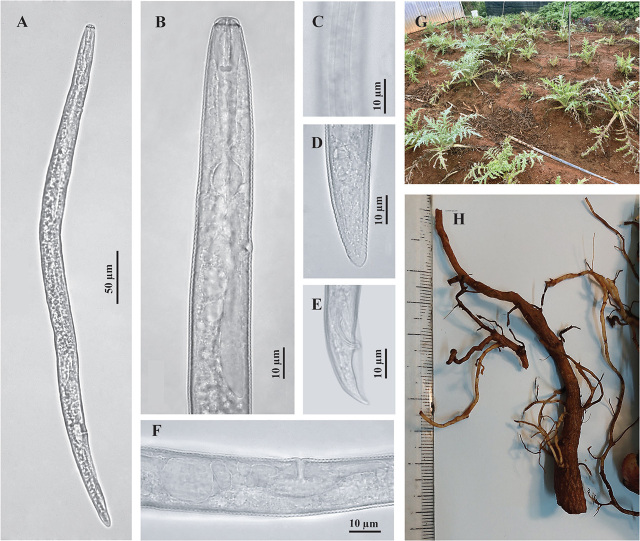
A–E: Light microscopy pictures of *Pratylenchus penetrans* from Vietnam. A–D, F: Female. A: Entire body, B: Anterior part of the body showing lip and pharynx region; C: Lateral field at mid-body; D: Tail region; F: Vulva region showing vulva and post-uterine sac. E: Tail region of the male; G, H: Symptoms of host plants; G: Aerial part; H: Underground part.

Body slightly slender ventrally when heat-relaxed (Fig. 1A). Lateral field with four lines at mid-body (Fig. 1C), becoming areolated posterior to vulva. Labial region with strong, conspicuous labial framework, slightly offset from body, low, flat anteriorly with three annuli (Fig. 1B). Robust stylet with rounded basal knobs. Pharyngeal gland overlapping intestine ventrally in a lobe *ca* 2 body diam long. Excretory pore opposite pharyngo-intestinal junction, located posterior to hemizonid. Hemizonid occupying *ca* two body annuli (Fig. 1B). Post-uterine sac short, *ca* 2 body diam at anus (Fig. 1F). Spermatheca spherical, full of sperm (Fig. 1F). Tail conical with a rounded and smooth tail tip (Fig. 1D).

#### Male

Slightly smaller than female but similar in form except for sexual features. Lateral fields with four lines at mid-body, ending on bursa (Fig. 1E). Spicules paired, slender, slightly ventrally arcuate; gubernaculum small and simple. Tail conical, bent on ventral side, enveloped by a peloderan bursa (Fig. 1E).

### Measurements

All measurements are presented in Table 1.

**Table 1.** Measurements of *Pratylenchus penetrans* from artichokes in Vietnam. All measurements are in μm (except for ratio) and in the form: mean ± s.d. (range)

**Characters**	***Pratylenchus penetrans* (Cobb, 1917; Filipjev & Schuurmans Stekhoven, 1941)**	
**Sex**	**Female**	**Male**
n	10	10
a	24 ± 2.1 (21–27)	27 ± 1.8 (24–30)
b	6.2 ± 0.2 (5.9–6.5)	5.7 ± 0.4 (5.3–6.2)
b′	3.9 ± 0.1 (3.7–4.1)	3.8 ± 0.2 (3.4–4.2)
c	17± 0.9 (15.7–18.8)	18.1 ± 1.4 (16.5–21)
c′	2.3 ± 0.1 (2.1–2.5)	2.2 ± 0.1 (2–2.4)
o	17.4 ± 1.6 (15.8–20)	18.9 ± 2.1 (15.1–22)
Body length (L)	502 ± 23 (446–528)	441 ± 19 (419–474)
V	80 ±1.1 (79–83)	–
Stylet length	15.8 ± 0.6 (15–16.8)	14.9 ± 0.5 (14.1–16)
Dorsal gland opening from stylet base (DGO)	2.8 ± 0.2 (2.5–3.1)	2.8 ± 0.3 (2.3–3.1)
Anterior end to valve of median bulb	53 ± 2.1 (50–57)	50 ± 2 (48–53)
Anterior end to nerve ring	65 ± 2.7 (61–69)	62 ± 3.1 (58–67)
Anterior end to secretory-excretory pore	81 ± 4.6 (71–88)	73 ± 3.4 (69–78)
Anterior end to pharyngo-intestinal junction	81 ± 4.5 (71–87)	78 ± 5.1 (72–89)
Anterior end to end of pharyngeal gland	129 ± 4 (121–136)	117 ± 5.6 (110–129)
Pharyngeal gland overlap	48 ± 3.2 (43–52)	39 ± 4.4 (33–49)
Max body diam.	21 ± 1.7 (18.7–23)	16.6 ± 1 (15.1–18)
Vulval body diam.	19 ± 1.4 (17–21.4)	–
Post-uterine sac (PUS)	26 ± 3.5 (17.7–29)	–
Anal body diam.	13.1 ± 1.1 (11.9–14.9)	11 ± 0.6 (10.4–12.4)
Tail length	30 ± 2.1 (27–33)	24 ± 1.9 (22–27)
Tail annuli at ventral side	23 ± 1.5 (21–25)	–
Spicule length	–	15.2 ± 0.9 (13.6–16.3)
Gubernaculum length	–	4.3 ± 0.3 (3.7–4.9)
PUS/Vulval body diam	1.4 ± 0.2 (1–1.6)	–

### Molecular characterization

#### Characterization of D2-D3 of 28S rRNA region

**Figure 2.**
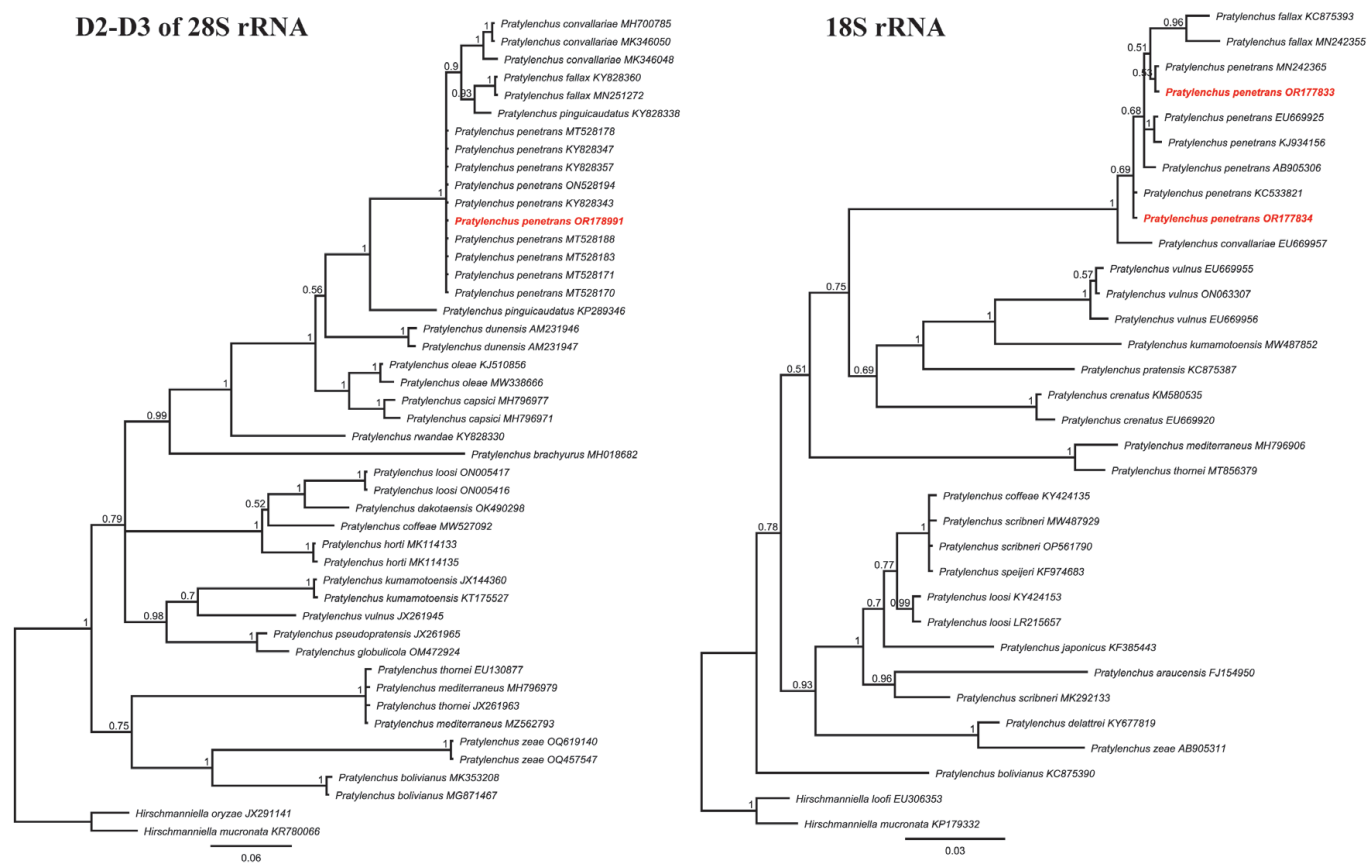
Bayesian phylogenetic tree of *Pratylenchus* species generated from 18S and D2D3 of 28S rRNA genes using the HKY+G+I and GTR+G+I models, respectively. Posterior Probability support was given next to each node. The sequences from this study were marked by red and bold.

The D2-D3 sequence of *P. penetrans* from Vietnam was 624 bp long (accession number: OR178991). This sequence is 100% identical to other sequences of *P. penetrans* from GenBank (accession number: MT528178, KY828347, KY828357, ON528194, KY828343, MT528188, MT528183, MT528171, MT528170). The D2-D3 sequences of *P. penetrans*, including those from Vietnam, are most similar to the sequences of *P. convallariae* (accession number: MK346048, MK346050, and MH700785) with 96.3–96.6% similarity (21–23 bp difference). All sequences of *P. penetrans*, *P. convallariae* (MH70085, MK346050, MK346048), *P. fallax* (KY828360, MN251272), and *P. pinguicaudatus* (KY828338) were placed in a single clade with maximal Posterior Probability support (Fig. 2).

#### Characterization of 18S rRNA region

Two 18S sequences of *P. penetrans* from Vietnam were obtained with a length of 939 bp (accession number: OR177833, OR177834). These sequences are 99.5–100% similar to other sequences of *P. penetrans* in GenBank (0–4 bp difference), while these sequences can be differentiated from the sequence of the closest species, *P. convallariae* (accession number: EU669957), by 12–16 bp (98–98.2% similarity). Two 18S sequences of *P. penetrans* from Vietnam and other sequences of *P. penetrans* (MN242365, EU669925, KJ934156, AB905306, KC533821), *P. convallariae* (EU9957), and *P. fallax* (KC875393, MN242355) formed the highest supported clade (1 PP) (Fig. 2).

#### Host symptoms

With the infestation of *Pratylenchus penetrans*, artichoke plants were stunted and showed yellowing leaves following a patchy pattern in the field. Root samples of infested host plants showed dark necroses along the roots.

## Discussion

Our research expands the understanding of the geographical distribution and host range of *Pratylenchus penetrans* by uncovering the first evidence of its infestation of artichokes in Vietnam. The morphological and morphometric characterizations of *Pratylenchus penetrans* from artichokes in Vietnam agree with the original and other descriptions of this species (Castillo & Vovlas, 2007). However, Janssen *et al.* (2017b) noted that species within the *Penetrans* group are morphologically very closely related, and there exists a mislabeled sequence of *P. penetrans* in GenBank. Consequently, to avoid misidentification of *Pratylenchus* species in general, the combination of morphological, morphometric, and molecular characterizations is needed. Molecular analyses in this study are also congruent with morphological observations, indicating our nematode population to be conspecific with *P. penetrans*. Our study provides molecular data of 18S and D2D3 of 28S rRNA regions of *P. penetrans* for the first time in Vietnam that were unequivocally linked with detailed morphological data to ensure accurate identification of this nematode species.

Previous reports from other countries have highlighted the detrimental impact of *P. penetrans* on artichokes and other crops (Castillo & Vovlas, 2007; Caubel *et al.*, 1975; Rossi & Monteiro, 2001; Vovlas & Roca, 1981). Compared to these reports, the average density of 50 nematodes/100g of soil in this study denotes a significant infestation and suggests the possibility of serious harm to Vietnam’s artichoke yields (Coosemans, 1975; Miller, 1975; Nyczepir & Becker, 1998; Olthof *et al.*, 1973). The presence of this nematode in 64.7% of the examined samples further emphasizes the severity of *P. penetrans* infestation in the investigated regions. Further evidence of *Pratylenchus penetrans*’ destructive capacity on artichoke plants in Vietnam are the observed symptoms, which include yellowing leaves, reduced growth, and root necrosis. These results raise concerns regarding the potential damage that *Pratylenchus penetrans* infection may cause for artichoke growers in Vietnam. Therefore, appropriate control strategies are highly recommended to mitigate the impact of *Pratylenchus penetrans* on artichoke cultivation in Vietnam.
